# An Overview and Perspectives on the Materials Genome Initiative-Based Asphalt Mix Design Framework

**DOI:** 10.3390/ma19132896

**Published:** 2026-07-06

**Authors:** Jian Liu, Zhen Wang, Fanijo Ebenezer, Linbing Wang

**Affiliations:** 1 School of Environmental, Civil, Agricultural and Mechanical Engineering, University of Georgia, Athens, GA 30602, USA; jian.liu1@uga.edu (J.L.); zhen.wang4@uga.edu (Z.W.); 2 School of Building Construction, Georgia Institute of Technology, Atlanta, GA 30332, USA; ebenfanijo@gatech.edu

**Keywords:** Materials Genome Initiative, asphalt mix design, asphalt binder, aggregate, laboratory testing, numerical simulation, artificial intelligence, data-driven

## Abstract

Traditional asphalt mix design approaches are trial-and-error processes and are limited in their ability to find high-performance asphalt mixtures to ensure long-term highway durability. The Materials Genome Initiative (MGI) launched in 2011 is designed to accelerate the development of advanced materials by integrating experiments, computational modeling, and big data analytics and has achieved remarkable progress in other materials design, which demonstrates strong potential for applying the MGI framework to optimize asphalt mix design. This study systematically reviews the application prospects of MGI methodologies in asphalt mixture design. Specifically, it first reviews recent progress in MGI for materials design and summarizes current research efforts to optimize asphalt mix design using traditional laboratory testing, numerical simulations (e.g., finite element method and discrete element method), and artificial intelligence while identifying their respective limitations. To overcome the limitations of each pillar, it is necessary to integrate these individual modules into a comprehensive MGI-based asphalt mix design framework. Furthermore, this study reviews the research on advanced experimental techniques for the multiscale characterization of asphalt binders and aggregates and defines the asphalt mixture genome, including binder-related genes, aggregate-related genes, and compaction-related genes. Finally, the specific steps for developing an MGI-based asphalt mix design framework are elaborated.

## 1. Introduction

In the U.S., approximately 94% of roads are surfaced with asphalt mixtures. Therefore, asphalt mixtures play a critical role in determining the durability and service life of pavements. Designing high-performance asphalt concrete is thus essential for ensuring desirable pavement longevity. Over the past few decades, researchers worldwide have made efforts to develop various asphalt mix design methods. Currently, widely adopted approaches, including the Marshall mix design method, the Superpave mix design method, and the more recently proposed balanced mix design, are primarily based on empirical trial-and-error processes and repeated laboratory testing. However, these methods are time- and resource-intensive. For example, completing a Superpave mix design of one mixture typically requires approximately 7.5 business days [1]. In addition, performance tests commonly used in BMD, such as the Hamburg Wheel Tracking Test (HWTT) and the indirect tension asphalt cracking test (IDEAL-CT), require approximately 5 days and 1 day, respectively, for one mixture [2]. Furthermore, because these methods rely on iterative trial-and-error procedures, their ability to efficiently explore the full mixture design space is limited, often resulting in suboptimal design solutions. As a result, they may overlook materials with superior performance that can simultaneously resist key degradation mechanisms, including permanent deformation, fatigue, abrasion, cracking, and moisture damage. Therefore, it is hard for traditional asphalt mixture design methods to meet the demands of high-durability pavement, highlighting the urgent need for a new asphalt mix design framework.

The Material Genome Initiative (MGI) was launched in 2011 in the U.S., with the goal of accelerating the discovery, development, and application of advanced materials. This initiative emphasizes the integration of three key technologies: high-throughput computational modeling, advanced experimental techniques, and data-driven approaches such as artificial intelligence (AI) and big data analytics. By leveraging these tools, the MGI aims to significantly shorten the time required for new material discovery to transition from laboratory investigation to real-world engineering implementation.

Tremendous asphalt mixture design research has already individually followed the three pillars of the MGI: advanced experimental testing for characterizing the physical and chemical properties of asphalt and aggregates, numerical simulations for modeling asphalt mixture behavior, and AI-based methods for performance prediction. However, each approach has its own limitations in optimizing mixture design. AI-based models can estimate asphalt mixture performance within seconds, but they often behave as “black boxes” and lack physical interpretability. Numerical simulations may require many hours or even several days to achieve satisfactory accuracy. Experimental mix design remains the most convincing and widely accepted; however, it is often time-consuming and labor-intensive. Therefore, it is promising to integrate the three pillars into a comprehensive MGI-based asphalt mixture design framework. Compared with a standalone AI-assisted asphalt mix design, the proposed MGI framework not only delivers physically interpretable results via numerical simulation but also incorporates experimental validation and data feedback through laboratory testing, which continuously enhances AI models’ predictive capability.

On the other hand, current researchers have only mentioned the MGI concept when investigating the effects of compositional factors on asphalt mixture properties. Specifically, they analogize the relationship between the physicochemical properties of composition components (e.g., asphalt or aggregates) and asphalt mixture performance (e.g., aggregate–asphalt interfacial adhesion strength) as a “gene–performance” relationship within the MGI approach [1,2]. However, there remains a lack of well-established frameworks or systematic procedures for fully leveraging MGI concepts in the design of high-performance asphalt mixtures.

**Figure 1:**
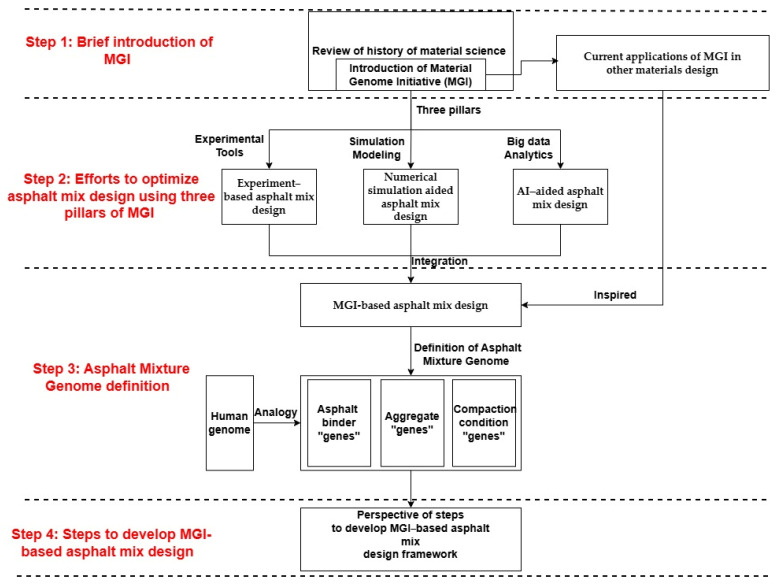
The overall framework of this study.

As discussed above, to better leverage the materials genome concept for optimizing asphalt mix design, this paper first reviews the MGI framework and its applications to other materials, then systematically examines current experimental, numerical simulation, and AI-based approaches in asphalt mix design, highlighting their limitations. Subsequently, the concept of the asphalt mixture genome is defined, including binder-related “genes”, aggregate-related “genes”, and compaction-related “genes”. Finally, this study elaborates the specific steps for developing the MGI-based asphalt mix design framework. A flowchart of this work is shown in Figure 1.

## 2. Material Genome Initiative (MGI)

### 2.1. Brief Introduction of Material Genome Initiative

**Figure 2:**
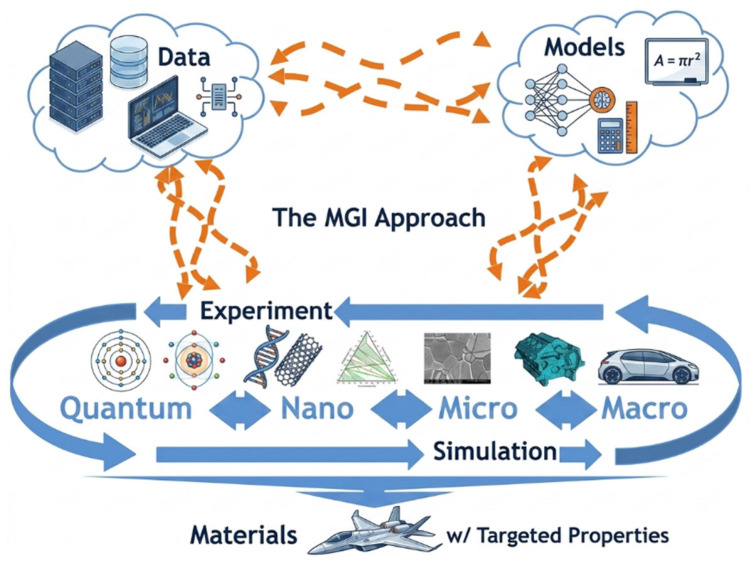
MGI framework linking data, experiment, and multiscale simulation.

The MGI is a U.S. government-led effort launched in 2011. Its core idea is to reduce the time and cost of materials innovation by combining experiments, computational modeling, and materials data infrastructure (databases and AI tools) (see Figure 2). The MGI allows researchers to strategically engineer materials with desired properties, effectively doubling the pace of innovation while significantly reducing the costs associated with moving new technologies from the laboratory to the market. The initiative also promotes a broader cultural shift in materials research: encouraging data sharing, open databases, and collaboration across academia, industry, and government, which brings faster innovation in areas such as batteries, alloys, polymers, and infrastructure materials, where improved performance can have large economic and societal impacts. By around 2014, the United States federal government had funded multiple research projects with a total budget exceeding 63 million dollars, supported by agencies including the National Science Foundation, the Department of Energy, and the Department of Defense. In Europe, the European Framework Programme 7 project Accelerated Metallurgy aimed to speed up the development of new alloys and metallurgical processes. In China, the Materials Genome Initiative strategic plan was launched in 2012 and further established in 2014 at Shanghai University [3].

### 2.2. Current Applications of MGI in Materials Design

Recently, the advent of AI has enabled the ongoing revolution of material science. As more innovative technologies related to data science allow more complex data to be processed and deeper correlations to be unveiled, the trend of applying data-driven methodologies to aid material design and discovery is emerging [4]. A lot of research efforts on predicting properties, screening, and optimizing materials have been made. In general, designing or discovering materials with the aim of machine learning (ML) or deep learning (DL) approaches consists of three key steps: (i) The first of these is a well-organized material database, and many comprehensive databases have been compiled in the past few decades, including inorganic crystal structures database (ICSD) [5,6], Open Quantum Materials Database [7], AFLOW [8], Materials Cloud [9], and MP [4]. (ii) The second involves the use of accurate predictive models for material properties that may exert their functions in different phases and steps of material design. In the past few decades, material properties that are measured from experiments or calculated by computational tools have been predicted by ML or DL methods with inputs of structural and elementary information, and successful examples include crystal structure prediction [10,11]; energies (e.g., molecular atomization energy [9,12], formation energy [13], energy gap prediction [14], and band gap energy [15,16,17]); the mechanical properties (e.g., Young modulus [18], hardness [19,20], tensile strength [21,22], and fatigue [23]) of metal alloys, glasses and steel; and thermal properties [15,24]. (iii) The third consists of methods for exploring the design space. Widely used methods include high-throughput screens on the known materials [25,26], generative models [27], and optimization algorithms [28,29]. Integrated workflows of materials design needs to put these three parts together and can be divided into three categories. The first workflow involves the virtual screening of materials from a large number of materials generated by a simple distribution or all possible combinations with various fundamental characteristics such as structures, electrical charge states [30], and ternary compositions [13] to check whether ML/DL-predicted physical and chemical properties can meet the corresponding criteria [30]. This philosophy has been applied to design compounds (A_x_B_y_C_z_) [13], catalysts [31], and metal–oxo moieties [30]. Designing new materials is also regarded as a Multi-objective problem because many criteria in design procedures need to be satisfied. The second framework involves using efficient optimization (e.g., genetic algorithm) to search materials that maximize a given property of interest [29,32,33] such as high strength, high thermal conductivity, and strong corrosion resistance. The third type of design is the inverse design, which starts at the desired properties and transforms discrete representation into continuous representations of the structural and elementary properties of materials by generative models (e.g., variational autoencoders), and then gradient-based optimization can be directly performed. This design method has been used to design organic molecules [34], solid-state materials [35], and crystalline porous materials [36]. Inspired by the progress of the MGI in other material systems, it is highly promising to extend MGI methodologies to asphalt mix design, particularly for optimizing mix design procedures using ML and DL.

## 3. Experimental, Simulation, and AI Methods for Asphalt Mixture

### 3.1. Experiment-Based Asphalt Mix Design in the U.S.

In the 20th century, the earlier widely acknowledged asphalt mix design method in the U.S. was the Hveem mix design, which is known for the Hveem stabilometer and kneading compactor [37]. Although the Hveem mix design can better simulate the densification characteristics of HMA in the real pavement, the test equipment is somewhat expensive and not portable. From the late 1940s to the early 1990s, the Marshall mix design method had gradually become the most widely used mix design method in the U.S. due to its inexpensive equipment and easily executable control/acceptance process and the greater attention paid to strength and air voids [38]. However, the Marshall mix design cannot fully evaluate the shear strength of HMA and does not address the desired properties which can match the local climate. To solve these issues, the Superpave mix design, a typical performance-based mix design methodology, was introduced under the Strategic Highway Research Program (SHRP) [39] and has been adopted by many states of the U.S. The Superpave mix design includes a binder specification system, the volumetric mix design, and the performance-based laboratory test procedure. With the increased use of recycled materials in asphalt concrete in recent decades, Superpave implementation has started to exhibit premature distresses such as cracking and raveling. In 2019, the U.S. Federal Highway Administration (FHWA) launched a Performance Engineered Pavements (PEP) vision that aims to focus more on long-term pavement performance by integrating pavement structural design, construction, and material acceptance. BMD is one of the key initiatives supporting the PEP [40]. Around this time, many states, such as Virginia [41,42], Georgia [43], Missouri [44,45], and Nebraska [46], have been exploring the feasibility of using the BMD to construct long-life asphalt pavements using local mixtures, traffic, and environmental conditions through a series of BMD-related research projects. To support this effort, numerous laboratory tests have been conducted to evaluate performance characteristics, including rutting resistance (e.g., HWTT and Asphalt Pavement Analyzer [APA]), cracking resistance (e.g., Illinois Flexibility Index Test (I-FIT) and IDEAL-CT), and moisture sensitivity tests (e.g., tensile strength ratio [TSR]). As a result, a vast amount of laboratory BMD data has been generated across various State Departments of Transportation (DOTs) and agencies. However, this data has yet to be systematically accumulated, integrated, and fully utilized. These performance-based mix design datasets form a critical foundation for the adoption of the MGI framework in asphalt mix design.

Regardless of whether the traditional mix design method or the increasingly studied BMD approach is used, both are trial-and-error processes and typically require multiple iterations. The entire procedure is time-consuming and resource-intensive. To address these challenges, research efforts to reduce the number of trial combinations for proportions and components have been ongoing. These efforts can be broadly classified into three key categories: optimized experimental procedures, numerical simulation-aided design, and AI-aided design.

### 3.2. Numerical Simulation-Aided Asphalt Mix Design

**Figure 3:**
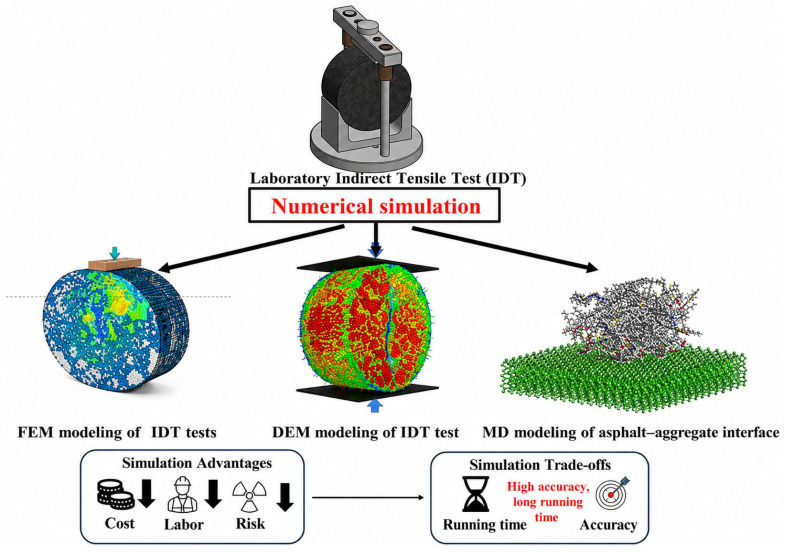
Application of FEM, DEM, and MD modeling in IDT tests of asphalt mixture.

Considering the complexity and time-consuming nature of performance testing in mix design procedures such as high-temperature stability, low-temperature crack and fatigue crack resistance, and durability tests, numerous researchers have applied various numerical simulation methods to conduct virtual testing as an alternative to traditional laboratory experiments at different scales. These methods include the finite element method (FEM), the discrete element method (DEM), and molecular dynamics (MD). Specifically, the FEM is widely used for simulating the macroscopic mechanical behavior of asphalt mixtures by incorporating accurate constitutive models, making it easier to analyze stress distribution, strain response, and deformation under various loading conditions [47,48]. The DEM is particularly effective for analyzing microscale interactions among mixture components and understanding the internal structure of asphalt mixtures [49,50]. Meanwhile, MD complements both the DEM and FEM by simulating the behavior of asphalt binder molecules at the atomic and molecular scales, providing valuable insights into fundamental material properties [51,52]. Figure 3 shows an example using numerical simulations to perform indirect tensile (IDT) tests.

Additionally, a digital twin for asphalt mixtures can be formed by integrating numerical simulation to virtually represent the entire asphalt mix design process [53]. However, few studies have integrated numerical simulations of performance tests into a mix design procedure, which is a key concern for related agencies or contractors. The authors’ team developed a framework to determine optimal asphalt content by correlating different asphalt contents with the DEM-based simulated compression test results of corresponding digital asphalt mixture samples [54], and this approach still follows the trial-and-error philosophy of traditional asphalt mixture design. On the other hand, although numerical simulations are lower in cost, require less labor, and have fewer risks compared to laboratory tests, executing complex performance tests via simulation can still be time-intensive, often taking hours or even days for a single test. Conducting multiple iterations for different trial mixture compositions would require an extensive number of simulations, making the process highly time-consuming. Therefore, relying solely on computational simulations to fully implement the asphalt mix design process is not yet a practical solution.

### 3.3. AI-Aided Asphalt Mix Design

#### 3.3.1. ML and DL for Asphalt Concrete Research

**Figure 4:**
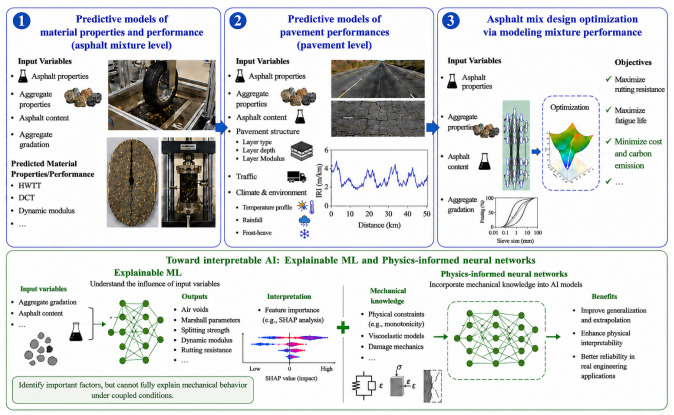
Overview of ML/DL approaches in asphalt mixture research.

The MGI approach emphasized the ability of data-driven computation to accelerate the development of advanced materials, which symbolized the beginning of the shift in materials design from traditional experiment-based empirical design to AI-based intelligent design [55]. As mentioned in Section 2, the remarkable progress in material design through ML/DL approaches has sparked significant interest in its application to asphalt concrete research (see Figure 4), making it a rapidly growing research area. Current research in this field can be categorized into three key themes: (1) predictive models of the material properties and performance (e.g., HWTT [56,57], Disk-shaped Compact Tension (DCT), and dynamic modulus [58,59]) of asphalt mixtures; (2) predictive models of pavement performance including rut depth [60,61], cracking [62,63], and the International Roughness Index (IRI) [64,65]; and (3) asphalt mix design optimization by the combination of ML-based mixture performance prediction [66,67] and metaheuristic algorithms (e.g., genetic algorithm) [68]. However, a major drawback of ML/DL approaches is that they function as “black-box” models, making accurate predictions without explaining the underlying physical relationships between mixture composition, material properties, and pavement performance.

#### 3.3.2. Explainable ML and Physics-Informed Neural Networks for Asphalt Concrete Research

In order to reflect the influence of asphalt mixture composition-based input variables, such as aggregate gradation and asphalt content, on predicted asphalt mixture properties, many researchers have applied explainable ML techniques to predict air voids [69], Marshall parameters [70], splitting strength [71], dynamic modulus [72], and asphalt mixture performance indicators such as rutting resistance. Most existing studies mainly use SHAP importance analysis to evaluate the importance of input variables, such as aggregate gradation and asphalt content. However, merely understanding the importance of input features cannot fully explain the mechanical behavior of asphalt mixtures under coupled loading, temperature, and moisture conditions. To improve the physical interpretability of AI-based predictions, especially from the perspective of mechanical theory, many researchers have incorporated various mechanical model-related loss terms into the loss functions of neural networks algorithms, which are commonly referred to as physics-informed neural networks, which aim to improve the generalization ability and extensibility of predicting asphalt mixture and pavement performance. For example, some studies introduced physical constraints, such as enforcing a monotonic increase in rut depth with increasing temperature and number of wheel passes, to predict rut depth [57]. Other studies introduced viscoelastic damage models to calculate stress and strain to calculate the loss when predicting Marshall stability and flow [73], while some incorporated viscoelastic continuum damage mechanics to predict the fatigue life in four-point bending tests [74]. However, the failure behavior of asphalt mixtures is a highly complex three-dimensional process. Existing physics-informed neural network approaches mainly introduce simplified one-dimensional constitutive models, which are still insufficient to fully describe such complex failure mechanisms. This still raises concerns about their reliability, especially in actual engineering applications.

### 3.4. Integration of MGI’s Three Pillars

As illustrated above, many researchers have conducted extensive work on the three fundamental tools of the MGI, including experiments, AI-powered data analytics, and numerical simulation, to evaluate asphalt mixture performance and optimize asphalt mix design. However, each approach has its own strengths and limitations.

Numerical simulation approaches (e.g., FEM, DEM, and MD) generally provide stronger physical interpretability because they are based on established material constitutive models. However, these methods still require laboratory testing (e.g., dynamic modulus tests) to obtain model parameters (e.g., viscoelastic parameters such as the Prony series coefficients). In addition, extensive model calibration is typically required through comparisons between simulation results and laboratory or field measurements. Numerical simulations can also be computationally demanding. Furthermore, when the asphalt mixture type, material source, or environmental conditions change, the constitutive model parameters often need to be recalibrated, and the validation process must be repeated.

In contrast, for AI-based approaches, a substantial amount of effort is devoted to collecting and preparing representative training datasets. The computational cost is primarily associated with model training and depends on both the complexity of the AI model and the size of the training dataset. During the training process, AI models are typically validated using independent datasets to evaluate their predictive performance. Once trained, AI-based models can rapidly predict material properties and pavement performance within seconds, making them computationally efficient for large-scale design optimization. However, compared with numerical simulation methods, AI-based approaches generally provide lower physical interpretability.

On the other hand, experimental mix design remains the most convincing approach for related agencies, as it directly measures material performance but is often time-consuming and labor-intensive. To overcome the limitations of each pillar, it is crucial to integrate experiments, big data analytics, and modeling into a comprehensive MGI mix design approach for asphalt mixtures.

## 4. Determination of Asphalt Mixture Genome

### 4.1. Human Genome-Inspired Analogy for Asphalt Mixture Genome

**Figure 5:**
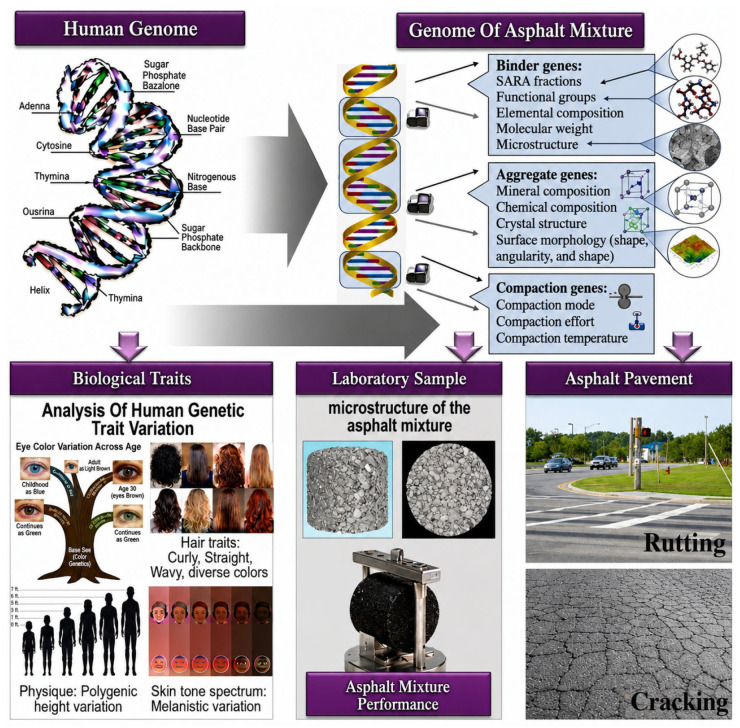
From human genome to asphalt mixture genome.

The materials genome methodology is analogous to the human genome. In humans, observable traits, such as hair color, eye shape, skin tone, and personality, are mainly determined by the human genome (see Figure 5). Therefore, there is an intrinsic relationship between genetic code and human traits. Similarly, in the field of materials science, extensive research has been conducted to establish relationships between the fundamental building blocks of materials, such as molecules and functional groups, and their properties and functions. By analogy to the human genome, these basic component units, along with their associated characterization or properties, can be regarded as the “genes” of a material. In other words, the complete set of these characteristic genes that collectively influence material performance is referred to as the “materials genome”. Therefore, we can define the “genes” of asphalt mixtures, a typical pavement material, as follows. Table 1 illustrates the analogy between the human genome and the asphalt mixture genome.

- Fundamental building blocks include atoms, molecules, and compounds, along with their physical and chemical properties and microstructures of asphalt binder and aggregates at both the macroscopic and microscopic levels. For example, for asphalt binders, macroscopic properties that can be regarded as asphalt mixture “genes” include density, viscosity, and the complex modulus, while microscopic characteristics include functional groups, molecular weight distribution, and microstructure (e.g., bee structures observed by atomic force microscopy (AFM)). For aggregates, macroscopic properties can be thought as asphalt mixture “genes” including density, abrasion resistance, and absorption, while microscopic characteristics include mineral composition, crystal structure, and morphology.
- The engineering performance of asphalt mixtures also depends on the mesostructure formed by the interaction and spatial arrangement of mixture components, which is, to some extent, controlled by compaction conditions, such as compaction mode, compaction temperature, and compaction effort.

The microstructural and compositional characterization of asphalt binders and aggregates at the microscopic level is typically obtained using advanced material testing techniques. For example, Fourier Transform Infrared Spectroscopy (FTIR) can be used to identify functional groups and chemical bonds in asphalt binders. These microscopic characteristics more intrinsically reflect the fundamental mechanisms governing the behavior of asphalt materials and mixtures. We refer to these as first-level “genes”. However, obtaining numerical data about these microscopic characteristics to build an asphalt mixture genome platform remains challenging, even though it is the foundation of the materials genome framework because advanced material testing facilities are often expensive and not readily accessible. Research and industry practice in traditional Marshall and Superpave mix design have already brought a substantial amount of experimental data. The macroscopic properties of asphalt binders and aggregates (such as penetration, viscosity, and Dynamic Shear Rheometer (DSR) parameters) are relatively easy to obtain but have not been fully utilized. These parameters also significantly influence the properties and performance of asphalt mixtures. Therefore, we define them as second-level “genes”.

In order to demonstrate the feasibility of defining the genes of binders, aggregates, and compaction, the following literature review will show that numerous studies have already utilized various testing methods to characterize these genes and confirmed their significant effects on asphalt mixture performance.

### 4.2. Asphalt Binder “Genes”

**Figure 6:**
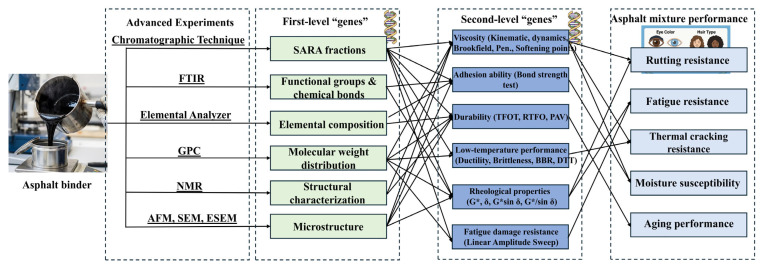
Asphalt binder genes and their relationship with asphalt mixture performance.

Asphalt is a solid or semi-solid mixture composed of high-molecular-weight hydrocarbons and their nonmetallic derivatives (such as oxygen, sulfur, and nitrogen compounds). As discussed in Section 4.1, the “genes” of asphalt refer to the intrinsic properties of asphalt binder that influence the physical and mechanical performance of asphalt mixtures. These characteristics span multiple scales, from the microscopic to the macroscopic level. At the macroscopic scale, asphalt binder properties are measured through common experimental tests, including viscosity, low-temperature performance, aging resistance, adhesion, rheological behavior, and mechanical properties. These are defined as second-level “genes”. At the microscopic scale, the “genes” refer to the chemical element composition, asphalt fractions, and microstructure of the asphalt binder. These are defined as first-level “genes”, which fundamentally affect the macroscopic second-level “genes” described above and ultimately influence the performance of asphalt mixtures. Figure 6 shows the relationship between asphalt first-level “genes” and second-level “genes” and how the second-level “genes” ultimately affect asphalt mixture performance. Table 2 presents the specific definition of “genes” regarding the microscopic and macroscopic properties of asphalt binder.

#### 4.2.1. SARA Fractions

**Figure 7:**
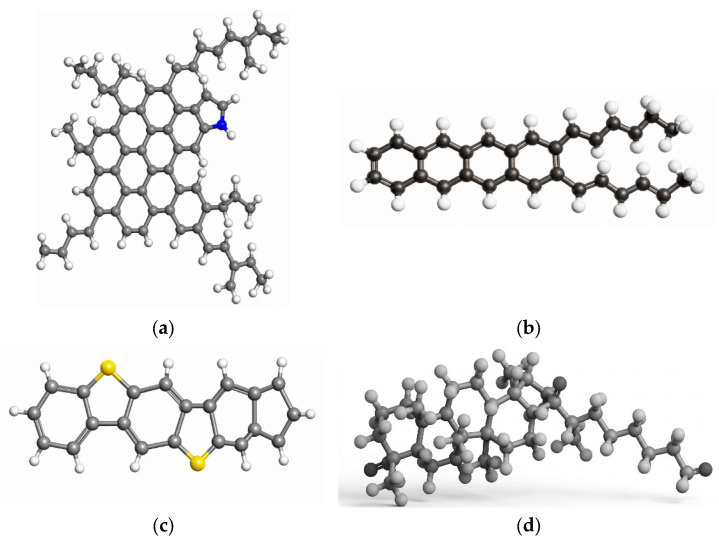
Typical molecular structure of SARA fractions of asphalt [98]: (**a**) aliphatic; (**b**) aromatics; (**c**) resin; (**d**) saturate.

Asphalt binder consists of thousands of hydrocarbons and non-hydrocarbon compounds. Based on physicochemical similarities among its components, asphalt is generally divided into four fractions: saturates (S), aromatics (A), resins (R), and asphaltenes (A) (see Figure 7) [77]. Numerous studies have demonstrated that these fractions have significant impact on the rheological, mechanical, and durability properties of asphalt binders. In general, heavier and more polar components, namely asphaltenes and resins, significantly increase the stiffness and complex modulus of asphalt binders, thereby improving elastic recovery and rutting resistance at high temperatures [90,91]. However, excessive asphaltene content may adversely affect low-temperature performance and increase cracking susceptibility. For example, Wang et al. [92] reported that variations in SARA fractions strongly influence low-temperature rheological properties such as creep stiffness, m-value, and glass transition temperature. SARA fractions also influence the fatigue performance of asphalt binder [93]. Studies by Jiang et al. [90,94] showed that increasing the content of asphaltenes and resins strengthens the colloidal structure of asphalt, which increases the complex modulus and affects fatigue resistance. Furthermore, the SARA fractions also affect adhesion and interfacial performance. Chen et al. [95] and Zhang et al. [96] found that asphaltenes and resins are positively correlated with binder–aggregate adhesion. In terms of aging performance, Guo et al. [97] reported that resins contain carbonyl functional groups that make them more susceptible to aging compared with other fractions.

#### 4.2.2. Functional Groups

Functional groups or chemical bonds, as important chemical constituents of asphalt binders, are typically identified using FTIR and have been shown to be strongly correlated with asphalt binder properties, especially aging-related properties. Previous studies have shown that oxidation during aging leads to the formation and accumulation of oxygen-containing functional groups, particularly carbonyl and sulfoxide groups [99,100]. Important rheological parameters, including the complex modulus, rutting factor, and creep stiffness, and filler–asphalt interaction [101] are also found to exhibit linear relationships with functional group indices such as the carbonyl index and sulfoxide index [102]. Furthermore, specific oxidation products, particularly carboxylic acids and ketones, have been identified as key components associated with the rutting and fatigue resistance of asphalt mixtures [100].

#### 4.2.3. Elemental Composition

The elemental composition of asphalt binder is also an important factor affecting its properties. The elemental composition is typically determined using an elemental analyzer (e.g., UNICUBE) [103]. The contents of C, N, and S have also been found to be strongly related to the surface free energy of asphalt [104], and the C/H ratio increases with the degree of asphalt aging [103].

#### 4.2.4. Molecular Weight Distribution

The molecular weight distributions (MWDs) of asphalt binder are fundamental compositional characteristics that strongly the mechanical and chemical properties of asphalt binder. Currently, the most popular method for measuring molecular weight distribution is Gel permeation chromatography (GPC), conducted by separating components based on their molecular size [105]. Many studies have investigated the relationship between MWD and asphalt binder performance. Wanli Ye et al. [106] reported that MWD polydispersity indices show strong correlations with rheological properties, including creep stiffness and fatigue resistance. Baek et al. [107] reported that the relative quantity of large molecular size is significantly correlated with the complex modulus (G*)/sin phase angle (δ) of the crumb rubber-modified binder. Regarding low-temperature properties, Elseifi et al. [108] observed that an increase in low-molecular-weight components leads to higher binder stiffness at low temperature.

Oxidation alters the molecular structure and composition of the binder, leading to an increase in high-molecular-weight molecules, consequently resulting in an increase in the viscosity of asphalt binder. The change in the physical and mechanical properties of asphalt binder can be explained by molecular weight distribution research [109]. Many studies have investigated the evolution of molecular weight distribution during the aging process of asphalt binders [109]. For example, it has been reported that small molecules transform into macromolecules during aging [110].

#### 4.2.5. Structural Characterization of Molecules and Compounds

Nuclear Magnetic Resonance (NMR) is a useful tool for recolonizing the structural characterization of the molecules and compounds of asphalt binder, such as methyl carbons, aromatic carbons, ring carbon, naphthenic carbons, and other parameters [111]. It is found that there is a strong correlation between NMR parameters and asphalt viscosity. NMR can also be used to measure the structure evolution of molecules and compounds to understand the asphalt aging process [112].

#### 4.2.6. Asphalt Binder Microstructure

Asphalt microstructure also influences the macroscopic properties of asphalt binders to a certain extent and can be regarded as a characteristic “gene” of the binder. Currently, the characterization of asphalt binder microstructure is primarily based on optical and electron microscopy techniques, including Scanning Electron Microscope (SEM), Environmental Scanning Electron Microscopy (ESEM), and Atomic Force Method (AFM).

##### Scanning Electron Microscope (SEM)

**Figure 8:**
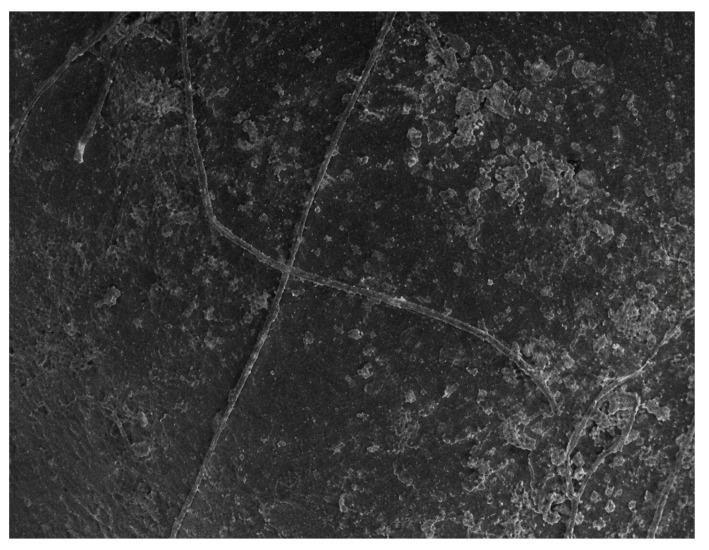
A typical SEM image of polyurethane-modified asphalt binder (100×) [115].

A scanning electron microscope (SEM) is an instrument that uses a focused beam of electrons, instead of light, to scan the surface of a material and produce high-resolution images of its microstructure. It can reveal surface morphology, texture, and composition at micro- to nanoscale resolution. A typical SEM image of modified asphalt binder is presented in Figure 8. Yang et al. [113] employed SEM to examine asphalt binders subjected to combined thermal oxidation and ultraviolet radiation aging. Their results showed that the raised surface structures gradually decreased with increasing aging, while more pronounced wrinkled features developed over time. In addition, Yao et al. [114] demonstrated that SEM is effective in evaluating the dispersion of additives, such as nanosilica material, within the asphalt binder matrix. However, SEM requires vacuum conditions and conductive coating, which may alter the binder surface and restrict observations to surface morphology.

##### Environmental Scanning Electron Microscopy (ESEM)

**Figure 9:**
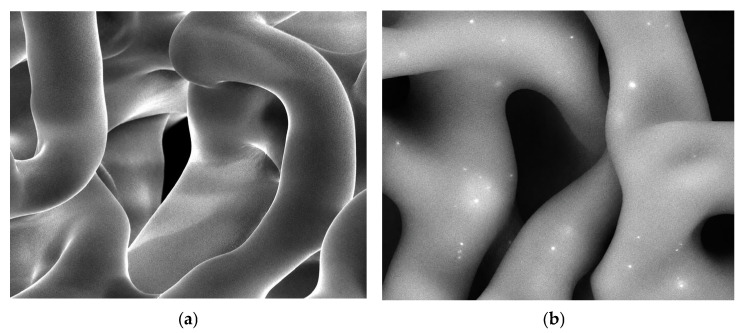
Typical ESEM microstructure images of asphalt binder: (**a**) virgin binder; (**b**) binder with additives (10 µm) [116].

Environmental scanning electron microscopy (ESEM) is an advanced form of SEM that enables the observation of materials under controlled environmental conditions, such as variable pressure and humidity. Unlike conventional SEM, ESEM does not require extensive sample preparation or conductive coating, allowing asphalt binders to be examined in a more natural state. Figure 9 shows the typical ESEM images of virgin asphalt binder and modified asphalt binder [116]. Previous studies have shown that the formation time and length of fibril structures under ESEM irradiation are associated with binder aging [117]. Lu et al. [118] reported that aging makes fibril formation more difficult, and there is a clear trend of decreasing in size and increasing in fibril formation time with aging. In addition, it is also found that fibril formation time and fibril area are well correlated with asphalt penetration and the softening point [119]. ESEM reduces sample preparation requirements and allows for observation under controlled environmental conditions; however, its resolution is generally lower than that of conventional SEM, and environmental settings may influence observed microstructural features.

##### Atomic Force Method (AFM)

**Figure 10:**
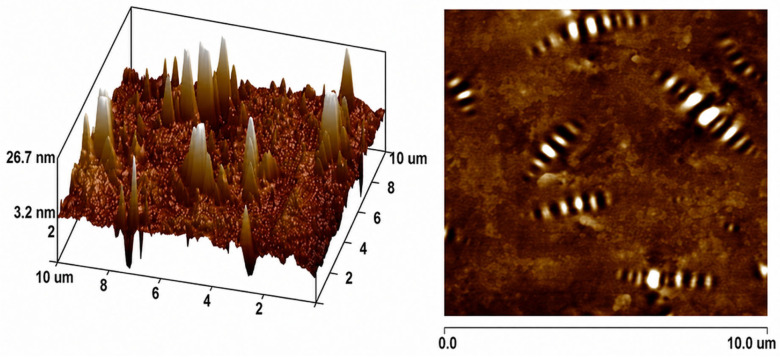
Typical images of AFM: three-dimensional height image and two-dimensional height image [126].

The AFM is capable of providing a topographic profile of the surface and achieving a resolution of the bitumen surface structure down to a few nanometers [120]. Generally, a typical morphology diagram of virgin asphalt binder presents a three-phase structure, including a “bee-like” dispersed phase, periphase and paraphase [121]. Loeber et al. [122] observed a “bee structure” in asphalt binder with AFM (see Figure 10). Pauli et al. [123] and Jager et al. [124] confirmed that “bee structures” were asphaltenes using AFM. McCarron et al. [125] found that temperature can influence the size and shape of bee structures and concluded that wax contributes to the formation of bee structures. With the addition of mineral filler particles, several significant changes can be observed regarding the morphological characteristics of asphalt binder, which can reflect the asphalt–filler adsorption mechanism.

Moreover, many studies have established relationships between bee structure parameters and the properties of asphalt binders. Specifically, the parameters of bee structures such as area, area ratio, the ratio of the long axis to the short axis, and roughness have been found to have a strong relationship with DSR parameters such as shear modulus and phase angle [127,128]. Additionally, a higher proportion of bee structures corresponds to lower penetration but a higher softening point, while increasing the roughness of the lightweight component region reduces ductility [126]. The authors of [129,130] show that the Derjaguin–Muller–Toporov (DMT) modulus measured by AFM and the asphalt dynamic modulus obtained from DSR show similar trends during asphalt binder aging and rejuvenation. AFM provides detailed nanoscale topographical and mechanical information; however, the testing process is relatively slow, only a small area can be scanned at a time, and the results are sensitive to testing conditions. In addition, the origin and interpretation of bee structures remain under debate.

### 4.3. Aggregate “Genes”

**Figure 11:**
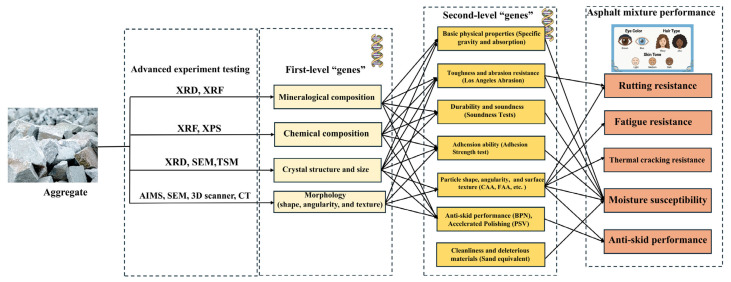
Aggregate genes and their relationship with asphalt mixture performance.

Aggregate-related “genes” refer to the intrinsic physical, mechanical, and morphological characteristics of aggregates that influence the performance of asphalt mixtures. Similarly to asphalt binders, these characteristics span from the microscopic level to the macroscopic engineering level. At the macroscopic scale (second-level “genes”), the aggregate characteristic genes include gradation, specific gravity, durability, abrasion resistance, shape and texture, and adhesion with asphalt binder. At the microscopic scale (first-level “genes”), the genetic characteristics of aggregates include mineral composition, chemical composition, crystal structure, acidity/basicity properties, and morphology, which fundamentally determine the macroscopic behavior of aggregates and ultimately affect the performance and durability of asphalt mixtures. Figure 11 shows the relationship between aggregate first-level “genes” and second-level “genes” and the effects of the second-level “genes” on specific asphalt mixture performance. Table 3 shows the aggregate “genes” defined based on aggregate multiscale characterization indices.

#### 4.3.1. Mineral Composition

Aggregate mineral composition is a fundamental genetic characteristic that significantly influences the properties of aggregates and asphalt mixture. These characteristics are typically determined using techniques such as X-ray diffraction (XRD) [154,155] and X-ray fluorescence (XRF) [156]. Aggregate mineral composition directly influences basic physical properties such as specific gravity and absorption. Zhang et al. [157] found that the moisture absorption of aggregates strongly depends on key minerals such as clay, anorthite, and calcite. Li et al. [158] found that the lithology of fine aggregates significantly affects the fatigue of asphalt mixtures.

The mineralogical composition also strongly affects the polishing and abrasion resistance of aggregates and skid resistance of asphalt pavement. Weak minerals may lead to degradation under environmental and traffic loading conditions. Malal Kane et al. [159] investigated the evolution of friction in different aggregates using the Wehner–Schulze apparatus under progressive polishing cycles. Their results showed that limestone, composed primarily of calcite with low hardness, is highly susceptible to polishing and exhibits the lowest friction levels. In contrast, aggregates such as graywacke and granite, which consist mainly of harder minerals like quartz and feldspars, are more resistant to polishing and maintain higher friction levels. Similarly, it was also found that the abrasion value is significantly related to the feldspar content, and the polished stone value of aggregates is positively correlated with calcite content and negatively correlated with quartz content [155].

Many studies have reported that the mineralogical composition exhibits significant influence on the adhesion property between bitumen and aggregate or asphalt mixture moisture susceptibility [156,160,161]. For example, Horgnies et al. [156] found that alkali feldspars in granite lead to weak adhesion by conducting peel tests on different aggregate–bitumen bonds. Based on similar experiment tests, Zhang et al. [157] reported that albite bonds well with bitumen in dry conditions and feldspar may also cause failure at the interface between bitumen and aggregates. Additionally, Fan et al. [128] studied the affinity between bitumen and six kinds of aggregates. Aggregates rich in nepheline, chlorite, pyroxene, and olivine show better resistance to moisture damage, while those rich in quartz, plagioclase, and calcite show poorer resistance. Cala et al. [162] demonstrated mafic aggregates (e.g., serpentinite) exhibiting better moisture resistance than felsic aggregates (e.g., quartzite and granodiorite) by pull-off tests under dry and moisture-conditioned states. The adhesion quality is also evaluated by moisture sensitivity tests. Bagampadde et al. [162] illustrated that aggregates with quartz and alkali feldspars show high stripping.

#### 4.3.2. Chemical Composition and Surface Acidity/Basicity

The chemical composition and surface acidity or basicity of aggregates are important factors affecting binder–aggregate interaction and moisture susceptibility. The chemical composition, such as SiO_2_, Al_2_O_3_, and Fe_2_O_3_, is always obtained by XRF. The atomic ratio of the aggregate such as C, O, Si, Ca, Na, and AI can be measured by X-ray Photoelectron Spectroscopy (XPS).

Most studies focus on investigating the effect of chemical composition on asphalt–aggregate adhesion and the moisture susceptibility of asphalt mixtures. In general, MgO in aggregates enhances resistance to moisture damage, whereas SiO_2_ tends to increase moisture susceptibility. Bagampadde et al. [163] investigated the influence of aggregate chemical composition on moisture sensitivity using tensile strength ratio tests on asphalt mixtures and found that mixtures containing aggregates with higher sodium and potassium exhibited relatively high moisture sensitivity. Wang et al. [164] quantitatively evaluated the adhesion between asphalt and eleven aggregates using a photoelectric colorimetric method. The results showed significant positive correlations between the adhesion rate and the contents of CaO and MgO, whereas SiO_2_ exhibited a negative correlation.

It is widely acknowledged that higher aggregate acidity (i.e., SiO_2_ content) tends to increase moisture susceptibility in hot mix asphalt, whereas higher aggregate basicity (i.e., calcium carbonate, CaCO_3_ content) is associated with lower moisture susceptibility [165]. Cheng et al. [166] reported that siliceous aggregates, which are predominantly composed of SiO_2_, are generally acidic and exhibit weaker bonding with asphalt. In contrast, calcareous aggregates, such as limestone with higher CaCO_3_, are alkaline and tend to form stronger adhesive bonds due to favorable acid–base interactions with the binder.

#### 4.3.3. Crystal Structure and Size

The crystal structure and size of minerals within aggregates are important microstructural characteristics. The crystal structure of aggregates is typically obtained using X-ray diffraction (XRD), SEM, and thin section microscopy (TSM) [154]. The general crystal structure of the surface strongly affects aggregate surface properties. Therefore, most research has focused on the influence of aggregate crystal structures on binder–aggregate interactions. Wang et al. [167] investigated the effects of SiO_2_, Al_2_O_3_, and CaO crystals on binder–aggregate interactions under different conditions using molecular dynamic simulations and found that the interaction strength with the asphalt binder follows the order Al_2_O_3_ > CaO > quartz. Granite with larger mineral particles and smooth, glossy crystal faces tends to have poorer adhesion with bitumen [168]. The crystallinity of minerals also plays a significant role in determining aggregate properties. High-crystallinity minerals have stronger physical and chemical stability due to their well-ordered crystal structures, while low-crystallinity minerals are more amorphous and have weaker mechanical properties [169]. Additionally, crystal structures also affect aggregate polishing and abrasion resistance. For example, Al_2_O_3_ crystal structures [170] can help form a network skeleton structure, which is useful for reducing polishing and abrasion damage.

#### 4.3.4. Aggregate Morphology

**Figure 12:**
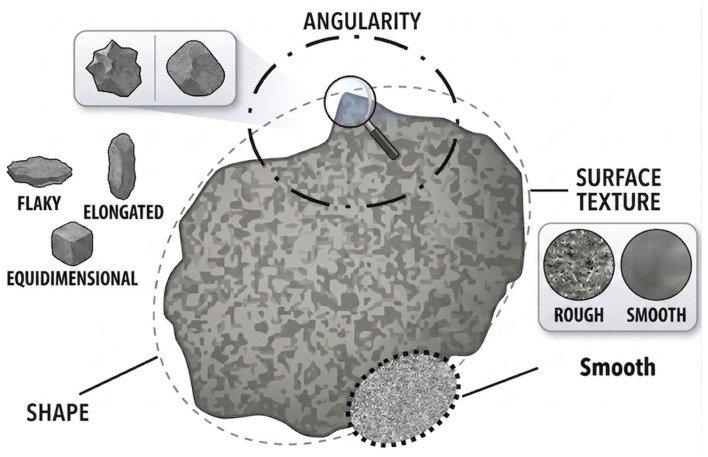
Description of shape, angularity, and texture of aggregate particles.

Aggregates in asphalt mixtures are typically obtained by crushing rocks from quarries and processing them into particles with various shapes. The morphological characteristics of aggregates, which are typically described using specific indicators, are generally divided into three levels: shape, angularity, and texture (see Figure 12). Establishing these morphological characteristic indices requires obtaining aggregate profiles using specialized devices. Common measure devices include the SEM, self-developed aggregate Image Measurement System (AIMS), 3D scanner (e.g., 3D blue-ray scanner [171]), X-ray CT [172], interferometry confocal 3D profiler [173], and Integrated LADAR Imaging System [174].

##### Aggregate Shape

The shape-based parameters of aggregates can be categorized into two-dimensional and three-dimensional parameters. Two-dimensional indices are typically obtained based on imaging measurement systems and include the form index [175], aspect ratio [176], axial coefficient [177], roundness [178], etc. These indices are always correlated to mixture performance. Eyad et al. [175] used imaging technology to obtain the form index to characterize the 2D features of aggregate shape and found that aggregate form indices are strongly correlated with rutting resistance. Wang et al. [177] employed the Aggregate Imaging Measurement System (AIMS) to quantify two-dimensional sphericity and further investigated the evolution of aggregate morphological characteristics during the Micro-Deval (MD) abrasion process. They also established the relationship between two-dimensional sphericity and aggregate polishing and abrasion resistance. Dharavath et al. [178] used the Automated Aggregate Imaging Measurement System (AIMS) to quantify aggregate roundness and found that the dynamic modulus and aggregate roundness exhibited similar ranking results for different aggregates.

3D shape characteristics are typically obtained using three-dimensional laser scanning [179] or X-ray computed tomography to capture detailed aggregate geometry. After obtaining the 3D spatial data of aggregates, 3D models of the particles are reconstructed. Furthermore, many researchers have proposed various 3D metrics to evaluate aggregate shape characteristics, including sphericity (derived from surface area and volume [180] or orthogonal dimensions [181]), shape factor, flat and elongated ratio, breadth-to-width ratio [182], and form indices based on Fourier series [183] and sphericity from harmonic analysis [184].

Jora et al. [185] investigated the effect of aggregate particle shape on the behavior of hot mix asphalt and found that rutting resistance (as indicated by flow number results) is strongly affected by aggregate sphericity. The dynamic modulus in compression also depends significantly on aggregate shape properties. The 3D shape characteristics of aggregates are also studied during the polishing process [186]. Wu et al. [187] studied the aggregate abrasion trend by analyzing 3D shape indices obtained from X-ray CT images of aggregates during the Micro-Deval test. Chen et al. [188] found that the shape and surface of aggregates influence asphalt workability during mixing. Among these, sphericity has the strongest effect, followed by surface texture and then angularity. Additionally, Hu et al. [189] also analyzed the influence of aggregate shape on the mix response to fatigue cracking and found that aggregate has a significant impact on mixture fatigue cracking.

##### Aggregate Angularity

Aggregate angularity describes how sharp, rough, or well-defined the edges and corners of aggregate particles are. Many researchers have focused on indices obtained from commonly used test methods (e.g., coarse aggregate angularity and fine aggregate angularity) to evaluate their effects on asphalt mixture performance [190]. For example, higher angularity improves rutting resistance by increasing aggregate interlocking [191], but it also leads to greater resistance to compaction [192]. In addition, higher angularity can enhance the mixture’s resistance to fatigue cracking because it requires more binder, which helps absorb energy and reduce damage [193].

Many researchers have proposed various angularity indices derived from high-precision measurements of aggregate morphology obtained using advanced measurement devices. A direct method for defining the angularity index is to use polygonal fitting to approximate aggregate angularity [194]. In addition, parameters derived from the Fourier series of the particle profile can be used to characterize aggregate angularity [195]. Using the FTI system to capture aggregate images, Sun et al. [173] also applied a two-dimensional Fourier transform method to develop an angularity factor (AF). Other mathematically based angularity indices include Gradient Angularity [172], 3D angularity [172], the angularity index [185], and the angularity coefficient [196]. Based on the defined aggregate angularity indices, their relationships with asphalt mixture performance have also been investigated. Wang et al. [172] found that the correlation between the skid resistance of asphalt pavement (texture depth value and British pendulum test) and the 3D aggregate index is significant. Based on the 3D aggregate index obtained from CT images, Gao et al. [197] also investigated the effects of coarse aggregate angularity on the compaction performance of asphalt mixtures, and the results show that a decrease in coarse aggregate angularity results in a decrease in dynamic modulus. Cui et al. [198] also found that there are close linear relationships between the aggregate angularity index and asphalt–aggregate adhesive strength and rutting resistance (dynamic stability).

##### Aggregate Texture

Aggregate texture describes the roughness or surface irregularities of aggregate particles, including how smooth, coarse, or uneven the particle surface appears. As one category of aggregate morphology characterization, numerous researchers have proposed a variety of indices to characterize aggregate surface texture. Aggregate texture indices are always defined based on transform-based signal processing methods, such as wavelet analysis or Fourier series analysis, applied to imaging measurement data of aggregates. Cui et al. [198] proposed a texture index to characterize aggregate texture from parameters obtained using the 2D wavelet method on aggregate images. Wang et al. [195] first used a combination of Fourier series parameters derived from aggregate particle profiles to define a surface texture index. Huang et al. [199] also did similar work. Subsequently, Sun et al. [173] and Liu et al. [200] also defined the texture factor of aggregates based on signals in the frequency domain transformed from the spatial domain of images using a two-dimensional FTI image analysis system. Other proposed angularity indices include the fractal dimension [201], erosion dilation area ratio [202], aggregate dimensions [203], and the 3D surface index using spherical harmonic coefficients [204].

In addition to influencing the fundamental properties of aggregates, such as water absorption [205], surface texture primarily affects the friction and the adhesion between asphalt and the aggregates of asphalt mixtures. Eyad Masad et al. [206] investigated the effects of aggregate surface texture measured by an aggregate Imaging System (AIMS) on pavement skid resistance after different polishing intervals. Similarly, Lei et al. [207] also found that the defined aggregate texture indices (Ra and Rq) have a higher correlation with anti-skid performance. Additionally, Zhan et al. [208] used a random forest analysis method to investigate the aggregate property factors influencing pavement friction. The results show that the loss of texture due to the Micro-Deval (MD) polishing test contributes the most to pavement friction.

Huang et al. [199] studied the effect of the aggregate surface texture index of granite, diabase, basalt, and limestone, measured using an AIMS, on surface free energy (SFE), which quantifies the adhesion between aggregate and binder. They found that a higher surface texture index leads to higher SFE and established a reliable exponential relationship between the surface texture index and SFE. Similarly, Gong et al. [209] also investigated the effect of aggregate surface texture (roughness) observed by laser confocal microscopy and three-dimensional blue light scanning on the aggregate–asphalt interface across different temperatures. They concluded that either excessively rough or excessively smooth aggregates are appropriate for forming a stable aggregate–asphalt interfacial interaction zone. Wang et al. [164] defined the aggregate texture index (fractal dimensions) based on the SEM picture of aggregates. The results show that a significant positive correlation was found between adhesion strength measured by the boiling water test and fractal dimensions. Wu et al. [210] also calculated the fractal dimensions of five types of rocks from SEM images.

Recent research also emphasizes the importance of aggregate roughness in asphalt mixture adhesion and durability. Ge et al. [205] reported that the interfacial properties of asphalt mixtures are closely related to aggregate surface roughness structure because adhesion involves not only chemical bonding and electrostatic effects but also mechanical interlocking. Pan et al. [211] concluded that moderate roughness improves adhesion by increasing the effective contact area between surfaces, whereas overly rough surfaces can reduce adhesion because they prevent full contact. Aggregate texture also influences the mechanical performance of asphalt mixtures. Fletcher et al. [212] developed a new image analysis-based procedure to quantify aggregate surface texture for both fine and coarse aggregates and evaluated its relationship with asphalt mixture performance. The results show that aggregate surface texture has a strong correlation with resistance to permanent deformation.

### 4.4. Compaction Condition “Genes”

When asphalt binders, aggregates, and other additives with specific properties are provided, compaction conditions play a decisive role in governing the internal structure, as well as the volumetric and mechanical properties of asphalt mixtures. This is primarily achieved by controlling air void distribution, density, and the internal distribution of aggregates. The compaction conditions most frequently investigated include compaction effort, compaction mode, and compaction temperature, which are collectively defined as compaction “genes”.

A consistent finding across previous studies is that compaction effort influences the air void content and distribution in asphalt mixtures, further affecting the asphalt mixture properties. Lee et al. [213] showed that increasing gyratory compaction levels significantly reduces air voids in crumb rubber-modified asphalt mixtures, and higher air void contents lead to lower rutting resistance. Similar conclusions were reported by Masad et al. [214], who found that air void distribution strongly correlates with compaction effort, and mixtures with lower air void content exhibit a higher modulus. The field and laboratory evidence provided by Hu et al. [215] further confirmed a linear relationship between the air void ratio and compactness degree. Some studies have directly focused on the relationship between compaction effort and asphalt mixture performance. Zhang et al. [216] demonstrated that increasing compaction effort improves rutting resistance but may reduce fatigue and low-temperature cracking resistance. Wang et al. [217] further showed that higher compactness leads to smoother pavement surfaces and lower high-speed friction, which suggests that excessive compaction may negatively affect skid resistance.

In addition to compaction level, the compaction method also plays a significant role in determining asphalt mixture structure and properties. Jiang et al. [218] compared different compaction methods, including vertical vibration testing, Marshall compaction, and modified Marshall compaction, and found that vibration-based compaction produces specimens with better consistency to field cores and higher mechanical performance (such as stability, compressive strength, and shear strength) at the same density level. Yu et al. [219] also reported that gyratory compaction produces better air void uniformity and superior rutting performance compared to Marshall and roller compaction methods.

Temperature is another critical factor that directly affects mixture workability and compatibility in compaction parameters. Rahmat et al. [220] found that insufficient compaction temperature increases air voids and reduces Marshall stability, resilient modulus, and creep resistance in dense graded mixtures. Similarly, Gao et al. [221] reported that both Marshall stability and volumetric properties are highly sensitive to compaction temperature. Kim and Kang [222] also confirmed that compaction temperature significantly affects volumetric properties such as the maximum specific gravity in polymer-modified mixtures.

The mesostructure of an asphalt mixture can be viewed as the result of the synergistic interaction between the asphalt binder, aggregates, and compaction conditions. Consequently, these are the fundamental factors that determine the comprehensive state of the mixture’s internal structure and overall properties.

## 5. Steps to Develop MGI-Based Asphalt Mixture Design Framework

**Figure 13:**
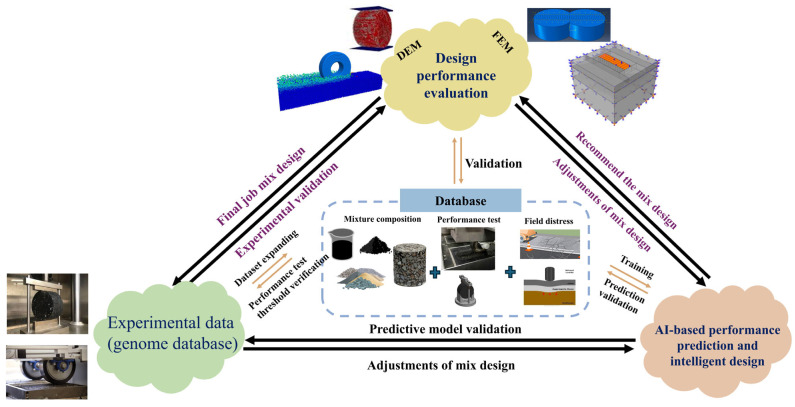
Integration of AI-assisted asphalt mix design, digital twin simulations, and experimental verification into MGI framework.

As illustrated above, many researchers have conducted extensive work on the three fundamental tools of the MGI, including experiments, AI-powered data analytics, and numerical simulation, to evaluate asphalt mixture performance and optimize asphalt mix design. However, each approach has its own limitations. To overcome the limitations of each pillar, we propose integrating these individual modules into a comprehensive MGI mix design framework for asphalt mixtures, as illustrated in Figure 13. Specifically, we propose the following:

1. The unified framework begins with the first module, focusing on data collection, filtering, cleaning, and storage to establish a robust asphalt mixture genome database that serves as the foundation for the MGI-based design framework. This database consists of datasets at multiple levels: (1) The first is the first-level genes of asphalt binders, such as SARA fractions; functional group indices; elemental composition indices such as the contents of C, N, and S; the percentage of large molecular size; NMR parameters; and the size and shape of bee structures observed using AFM. It also consists of the first-level genes of aggregates, such as mineral composition percentages (e.g., quartz, feldspar, and chlorite); chemical composition ratios such as SiO_2_, Al_2_O_3_, and Fe_2_O_3_; aggregate morphological properties including shape indices (sphericity or shape factor), angularity indices (angularity index), and aggregate indices (texture index); and compaction condition genes such as compaction effort, compaction mode, and compaction temperature [the testing methods for obtaining these indices can be seen in Table 1 and Table 2]. (2) Next are the second-level genes of asphalt and aggregates from numerous asphalt mix design data points, such as binder viscosity, ductility, creep stiffness, and complex modulus, as well as aggregate gradation, absorption, CAA, and FAA [the testing methods for obtaining these indices can be seen in Table 1 and Table 2]. (3) The third consists of asphalt mixture volumetric properties, such as air void and bulk density and asphalt mixture performance indices, such as rut depth in the HWTT and the CT Index in IDEAL-CT. (4) The fourth is represented by field performance records, as well as Quality Control (QC)/Quality Assurance (QA) data from plant production and field placement. The variables mentioned above can be collected from open databases (e.g., Long-Term Pavement Performance Program), published papers and reports, and data shared by related institutions or measured directly if experimental conditions allow. The first- and second-level genes, asphalt mixture performance indices, and QA/QC data from the same asphalt mixture will be stored in the same row of the asphalt mixture genome database. The variables mentioned above are intended to be collected as completely as possible when the corresponding data are available.
2. Next, the genome database is then used to develop ML/DL (e.g., random forest and physics-informed neural networks)-based predictive models for volumetric properties (e.g., air void), mixture performance indices (e.g., rut depth in HWTT), and field performance (e.g., rutting), using the selected asphalt mixture genome (first-level and second-level “genes”) and composition proportion as inputs. When selecting specific genes for modeling particular mixture performance, variables in the genome database with available and complete data are first selected. Feature engineering techniques, such as ML-based sensitivity analysis, are then applied to identify the significant genes from the entire genome database. To account for mixture genome variability such as aggregate variation in quarry source, mineralogical variability between regions, blending variability, and inter-laboratory variability, probabilistic ML and DL methods, which can explicitly incorporate variability in genome variables and quantify their effects on predicted performance, are also supposed to be used. After the predictive models are well trained, the mixture performance (e.g., rut depth in HWTT) of a trial composition can be estimated if the genome of that trial is known. With the trained predictive models, the candidate asphalt mixture designs are recommended using Multi-objective Optimization (MOO) techniques (e.g., Non-dominated Sorting Genetic Algorithm-II (NSGA-II) and reinforcement learning-based methods), considering a balance between rutting and cracking resistance, life-cycle cost, and environmental impact.
3. The recommended candidate mix designs are then evaluated in the second module through digital twin simulations, utilizing the numerical methods of the DEM and FEM to conduct virtual tests. These simulations cover a wide range of performance evaluations, including material properties such as Marshall stability and the dynamic modulus, as well as key performance metrics like rutting resistance, such as the HWTT, and cracking resistance, such asIDEAL-CT. Additionally, the digital twin framework enables the prediction of field distress evolutions, including rutting and cracking, under the climatic and traffic conditions specific to the location where the asphalt concrete will be placed. Notably, the database developed in the first module plays a critical role in calibrating and validating the digital twin models, ensuring that the virtual simulations accurately reflect real-world performance.
4. Finally, in the third module, the optimal asphalt content and gradation determined from the analysis of the digital twin simulation results are validated through real laboratory tests. These experimental tests not only confirm the accuracy of both the AI-based predictions and the simulation results but also contribute to expanding the dataset in the first module. If the test results from either the digital twin simulations or the real laboratory tests do not meet the required performance thresholds, feedback is provided to the AI-based prediction and mix design module. This feedback loop triggers adjustments to the AI model parameters and the MOO-based mix designs, enabling the generation of new mixture proportions. The process then iterates until the performance criteria are successfully met, establishing a continuous improvement loop for optimizing asphalt mix design.

In summary, this framework consists of three iterative stages: Computer-Aided (Generated) Mix Design, initially developed through a data-driven approach; the development of a digital twin for performance evaluation and mixture screening using rational modeling approaches; and the experimental verification and validation of the mix design and performance.

However, the success of the MGI-based asphalt mixture design framework strongly depends on the availability, quality, standardization, and completeness of the collected data. The established mixture genome database may face several challenges, including: (1) data heterogeneity caused by different equipment and data formats used across laboratories and agencies; (2) the lack of standardized testing and reporting protocols for certain material genes and performance indicators; (3) missing, incomplete, or imbalanced datasets; and (4) barriers to data sharing among agencies, laboratories, and industries because of proprietary restrictions and inconsistent database structures.

To mitigate these issues, several strategies are proposed. First, existing standardized testing protocols, including ASTM and AASHTO specifications, should be consistently followed when conducting tests on asphalt binders, aggregates, and asphalt mixture properties. Regarding testing methods for which no standardized protocols currently exist, related agencies and standardization organizations are encouraged to establish unified protocols, especially for measuring first-level gene variables such as the functional group indices of asphalt binders measured by FTIR and aggregate morphology indices measured using an AIMS. Second, data preprocessing techniques would be used, including normalization, outlier detection, and missing data imputation methods, such as mean/median imputation, k-nearest neighbor imputation, and ML-based imputation approaches. Third, establishing collaborative data-sharing platforms, such as LTPP databases, among DOTs, universities, and industry partners could gradually expand the asphalt mixture genome database. In these platforms, standardized data templates and reporting protocols can facilitate future data sharing and interoperability across institutions.

## 6. Conclusions

Traditional asphalt mix design approaches are trial-and-error processes and are not effective for designing high-performance mixtures. The MGI aims to accelerate materials design and discovery by integrating experiments, computational modeling, and big data analytics, and it has achieved significant progress in the field of materials science. Inspired by this progress, this study proposed leveraging the MGI concept to optimize asphalt mix design. Specifically, this study first summarized current research efforts to accelerate and optimize asphalt mix design using traditional laboratory testing (performance tests), numerical simulations (e.g., FEM and DEM), and AI (e.g., ML and DL). Subsequently, this study reviewed recent advances in high-end experimental techniques for the multiscale characterization of asphalt binders and aggregates. Based on these multiscale characterization indices, the asphalt mixture genome was defined, including binder-related genes, aggregate-related genes, and compaction-related genes.

The review results indicate that laboratory-based mix design procedures are time-consuming and resource-intensive. Meanwhile, ML/DL models can predict asphalt mixture performance within seconds; however, they often function as black boxes and lack physical interpretability, providing accurate predictions without explaining the underlying physical relationships. In contrast, numerical simulations are physically interpretable but often require several hours to be completed. To address these limitations, this study proposes an MGI-based asphalt mix design framework that integrates AI-assisted mix design, digital twin-based simulation modeling, and experimental verification and elaborates specific procedures for implementing the framework. The mix design framework is expected to be implemented as executable software to support its use in research and engineering practice.

## 7. Limitations and Suggestions for Future Work

Numerous studies have demonstrated that the mesostructure of asphalt mixtures, mainly including the spatial distribution of air voids and coarse aggregates, has a strong relationship with asphalt mixture performance. However, this study did not discuss in detail how these mesostructural characteristics can be regarded as mixture structural genes. Future research should further explore the integration of mesostructural features with the MGI concept.

In addition, the proposed MGI-based asphalt mix design framework is still at a conceptual stage. One of the major challenges remains the availability, quality, standardization, and completeness of a comprehensive genome database, which requires the development of a standardized database framework and data-sharing platform. Addressing these challenges will require substantial funding, government-level coordination, and collaboration among multiple research institutions and industry stakeholders, which will require more detailed and large-scale implementation plans.

In future research, we will first explore the feasibility of the proposed asphalt mixture design framework by establishing a small-scale asphalt mixture genome database and implementing the key steps of the proposed framework. These steps include developing predictive models for asphalt mixture properties and performance (e.g., rut depth of HWTT and fatigue life of the repeated flexural bending test), conducting virtual testing and design optimization using DEM simulations, and performing laboratory tests for performance validation. Considering the difficulty of obtaining first-level gene variables, the initial case study may primarily utilize second-level gene variables. Furthermore, a database containing approximately 2000 records should be sufficient for the initial implementation and validation of the framework.

## Figures and Tables

**Table 1:** Mapping human genome concepts to asphalt mixture genome components.

Concept	Asphalt Mixture Genome	Example Genes/Descriptors	Corresponding Outcomes	
Human Genome	Nucleotide Genes	DNA sequence, nucleotide composition, genetic variants, gene expression patterns	Biological traits (Eye color, hair characteristics, skin pigmentation, height, etc.) and physiological functions	
Asphalt Mixture Genome	Binder Genes	SARA fractions, functional groups, elemental composition, molecular weight, microstructure	Mixture rheological and mechanical properties	Asphalt mixture rutting, fatigue, and thermal cracking resistance; moisture susceptibility, and anti-skid performance
	Aggregate Genes	Mineral composition, chemical composition, crystal structure, surface morphology (shape, angularity, texture)	Aggregate skeleton and air void distribution	
	Compaction Genes	Compaction mode, compaction effort, compaction temperature	Asphalt mixture structure and mixture density	

**Table 2:** Asphalt binder “genes” from asphalt binder multiscale characterization indices.

First-Level “Genes” (Asphalt Binder Micro-Characterization) *	Second-Level “Genes” (Asphalt Binder Macroscopic Property) *		
	Property Indicator		Test Specification
SARA fractions (ASTM D4124 [75]) Molecular weight distributions (GPC) Microstructure [fibril structure dimension and formation time (ESEM) bee structures (AFM)] Structural characterization [Nuclear Magnetic Resonance (NMR)]	Viscosity	Kinematic Viscosity, mm^2^/s	ASTM D2170 [76,77]
		Dynamic Viscosity: Brookfield Viscosity, Pa·s	ASTM D4402 [78]
		Penetration Value, 0.1 mm	ASTM D5 [79]
		Softening Point, °C	ASTM D36 [80]
SARA fractions (ASTM D4124) Functional groups (FTIR) Elemental composition (elemental analyzer) Microstructure [bee structures (AFM)]	Adhesion ability	Asphalt Binder Bond Strength test, MPa	AASHTO TP 91 [81]
SARA fractions (ASTM D4124) Molecular weight distributions (GPC) Functional groups (FTIR) Elemental composition (elemental analyzer) Microstructure [bee raised surface structures (SEM) fibril structure dimension and formation time (ESEM) bee structures (AFM)] Structural characterization [Nuclear Magnetic Resonance (NMR)]	Asphalt Durability (Aging performance)	Thin-Film Oven Test (TFOT) (physical property changes, %)	ASTM D1754 [82]
		Rolling Thin-Film Oven Test (RTFO) (physical property changes, %)	ASTM D2872 [83]
		Pressure Aging Vessel (PAV) (physical property changes, %)	AASHTO R 28 [84]
SARA fractions (ASTM D4124) Molecular weight distributions (GPC)	Low-temperature performance	Ductility, cm	ASTM D113 [85]
		Bending Beam Rheometer (BBR) (creep stiffness, MPa; m-value)	AASHTO T 313 [86]
		Direct Tension Test (DTT) (failure strain)	AASHTO T 314 [87]
SARA fractions (ASTM D4124) Molecular weight distributions (GPC) Functional groups (FTIR) Microstructure [bee structures (AFM)]	Rheological properties (High-temperature and medium-temperature performance)	Dynamic Shear Rheometer (DSR) (complex modulus (G*); Phase angle (δ), G*sin δ, G*/sin δ)	AASHTO T 315 [88]
SARA fractions (ASTM D4124) Molecular weight distributions (GPC)	Fatigue damage resistance	Linear Amplitude Sweep	AASHTO T 391 [89]

* First-level “genes” in each row influence the second-level “genes” in the same row.

**Table 3:** Aggregate “genes” from aggregate multiscale characterization indices.

First-Level Genes (Aggregate Microscopic Characterization) *	Second-Level Genes (Aggregate Macroscopic Property) *		
	Macroscopic Property Indicator		Test Specification
N/A	Gradation and size	Gradation size distribution (%); NMAS	ASTM C136 [131]/AASHTO T27 [132]
Mineralogical composition (XRD and XRF), chemical composition (XRF and XPS), crystal structure (XRD, SEM, and TSM)	Basic physical properties	Specific gravity	AASHTO T 84/85 [133,134] and ASTM C 127/128 [135,136]
		Absorption	AASHTO T 84/85 [133,134] and ASTM C 127/128 [135,136]
Mineralogical composition (XRD and XRF), chemical composition (XRF and XPS), crystal structure (XRD, SEM, and TSM), morphology (AIMS, SEM, 3D scanner, interferometry profiler, Computed tomography (CT))	Toughness and abrasion resistance	Los Angeles Abrasion (Los Angeles abrasion loss, %)	AASHTO T 96 [137] or ASTM C 131 [138]
Mineralogical composition (XRD and XRF), chemical composition (XRF and XPS), crystal structure (XRD, SEM, and TSM)	Durability and soundness	Soundness tests (soundness loss, %)	ASTM C88 [139]/AASHTO T104 [140]
Mineralogical composition (XRD and XRF), chemical composition (XRF and XPS), crystal structure (XRD, SEM, and TSM), microstructure morphology (AIMS, SEM, 3D scanner, interferometry profiler, CT)	Interface adhesion	Asphalt–aggregate interface adhesion strength test, MPa	AASHTO TP 91 [81]
N/A	Cleanliness and deleterious materials	Clay lumps and friable particles, %	AASHTO T 112 [141] and ASTM C 142 [142]
		Sand equivalent, %	AASHTO T176 [143]
Microstructure morphology (AIMS, SEM, 3D scanner, interferometry profiler, CT)	Particle shape, angularity, and surface texture	Particle index, %	ASTM D 3398 [144]
		Flat and elongated particles, %	ASTM D 4791 [145]
		Coarse aggregate angularity (percentage of fracture in coarse aggregate, %)	AASHTO TP 61 [146]/ASTM D 5821 [147]
		Uncompacted/compacted void content of coarse aggregate, %	AASHTO TP 56 [148]/AASHTO T 19 [149]
		Fine aggregate angularity, %	AASHTO T 304 [150] or ASTM C 1252 [151]
Mineralogical composition (XRD and XRF), chemical composition (XRF and XPS), crystal structure (XRD, SEM, and TSM), microstructure morphology (AIMS, SEM, 3D scanner, interferometry profiler, CT)		Accelerated polishing of aggregates (polished stone value (PSV))	ASTM D3319 [152]
Mineralogical composition (XRD and XRF), chemical composition (XRF), crystal structure (XRD, SEM, and TSM), microstructure morphology (AIMS, SEM, 3D scanner, interferometry profiler, CT)	Anti-skid performance	British pendulum number (BPN)	ASTM E303-22 [153]

* First-level “genes” in each row influence the second-level “genes” in the same row.

## Data Availability

No new data were created or analyzed in this study. Data sharing is not applicable to this article.
